# Supplementary material for: A general approach for selection of epitope-directed binders to proteins
Source: Proc Natl Acad Sci U S A. 2024 Apr 29;121(19):e2317307121. doi: 10.1073/pnas.2317307121 (PMC11087759; doi:10.1073/pnas.2317307121)
Supplement: Supplementary file 1 — Appendix 01 (PDF) [file pnas.2317307121.sapp.pdf]

PNAS

Supporting Information for

A general approach for selection of epitope-directed binders to proteins

Jie Zhou[a, d], Chau Q. Le[a], Yun Zhang[a], James A. Wells[a, b, c, *]

[a]Department of Pharmaceutical Chemistry, University of California San Francisco, San Francisco, CA, 94158, USA.

[b]Chan Zuckerberg Biohub; San Francisco, CA, 94158, USA.

[c]Department of Cellular and Molecular Pharmacology, University of California San Francisco, San Francisco, CA, 94158, USA.

[d]Department of Radiation and Cellular Oncology & Department of Chemistry, University of Chicago, Chicago, IL, 60637, USA.

*Corresponding Author: James A. Wells

Email: jim.wells@ucsf.edu

Author Contributions: J.Z. and J.A.W. designed research; J.Z., C.Q.L., Y.Z., and J.A.W. performed research; J.Z., C.Q.L., and J.A.W. contributed new reagents/analytic tools; J.Z., C.Q.L., and J.A.W. analyzed data; and J.Z. and J.A.W. wrote the paper.

Competing Interest Statement: The authors claim no competing interest.

Classification: NA

Keywords: epitope-directed antibody; phage display; differential selection; proteolysis; cancer therapy

This PDF file includes:

SI Appendix, Figure 1 – 10
SI Appendix, Table 1

a

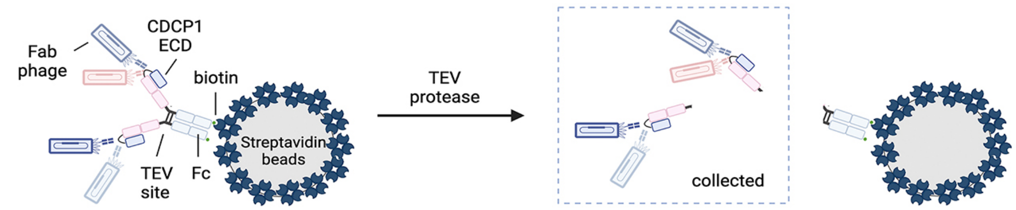

b

			fold of enrichment	
	negative	positive	Round 3	Round 4
selection campaign 1	CDCP1-Fc^{mutant1}	CDCP1-Fc^{wt}	1	10
selection campaign 2	CDCP1-Fc^{mutant2}	CDCP1-Fc^{wt}	1	100
selection campaign 3	CDCP1-Fc^{mutant3}	CDCP1-Fc^{wt}	1	100

c

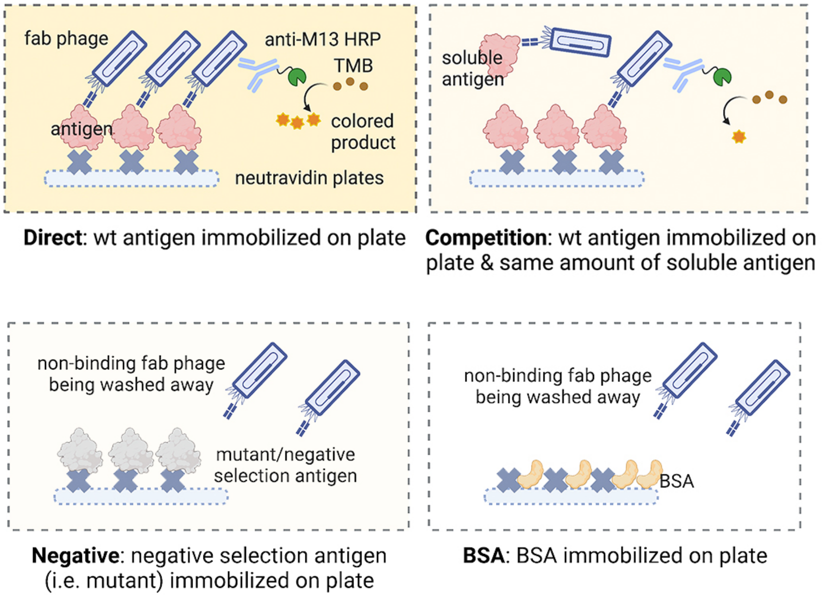

SI Appendix, Figure 1. (a) A catch-and-release strategy used for phage selection. (b) The enrichment was characterized by phage titer as a function of round of selection. (c) Schematic illustration of phage ELISA.

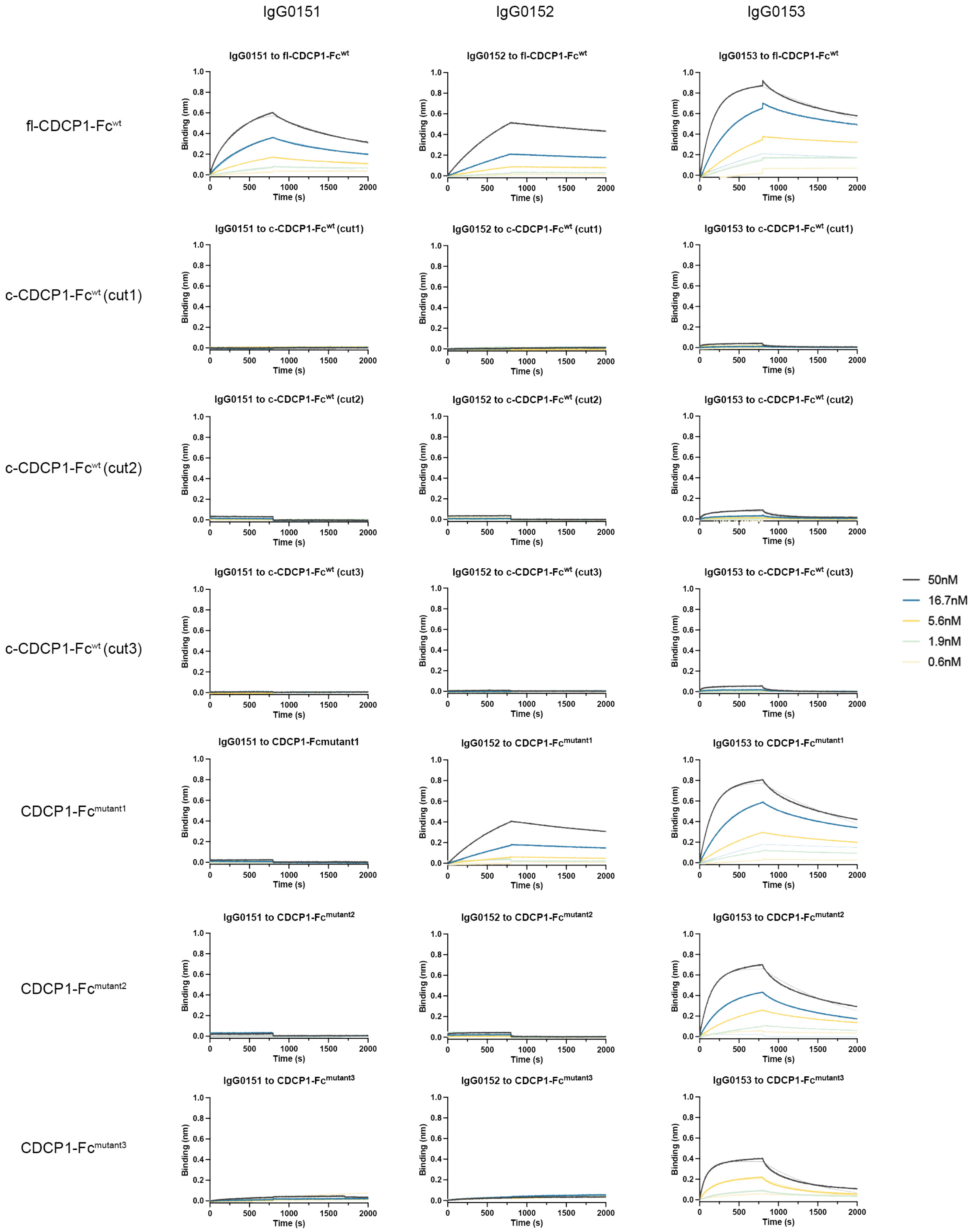
IgG0151
IgG0152
IgG0153
fl-CDCP1-Fc^wt
c-CDCP1-Fc^wt (cut1)
c-CDCP1-Fc^wt (cut2)
c-CDCP1-Fc^wt (cut3)
CDCP1-Fc^mutant1
CDCP1-Fc^mutant2
CDCP1-Fc^mutant3
IgG0151 to fl-CDCP1-Fc^wt
IgG0152 to fl-CDCP1-Fc^wt
IgG0153 to fl-CDCP1-Fc^wt
IgG0151 to c-CDCP1-Fc^wt (cut1)
IgG0152 to c-CDCP1-Fc^wt (cut1)
IgG0153 to c-CDCP1-Fc^wt (cut1)
IgG0151 to c-CDCP1-Fc^wt (cut2)
IgG0152 to c-CDCP1-Fc^wt (cut2)
IgG0153 to c-CDCP1-Fc^wt (cut2)
IgG0151 to c-CDCP1-Fc^wt (cut3)
IgG0152 to c-CDCP1-Fc^wt (cut3)
IgG0153 to c-CDCP1-Fc^wt (cut3)
IgG0151 to CDCP1-Fcmutant1
IgG0152 to CDCP1-Fc^mutant1
IgG0153 to CDCP1-Fc^mutant1
IgG0151 to CDCP1-Fc^mutant2
IgG0152 to CDCP1-Fc^mutant2
IgG0153 to CDCP1-Fc^mutant2
IgG0151 to CDCP1-Fc^mutant3
IgG0152 to CDCP1-Fc^mutant3
IgG0153 to CDCP1-Fc^mutant3
Binding (nm)
Time (s)
0.0
0.2
0.4
0.6
0.8
1.0
0
500
1000
1500
2000
50nM
16.7nM
5.6nM
1.9nM
0.6nM

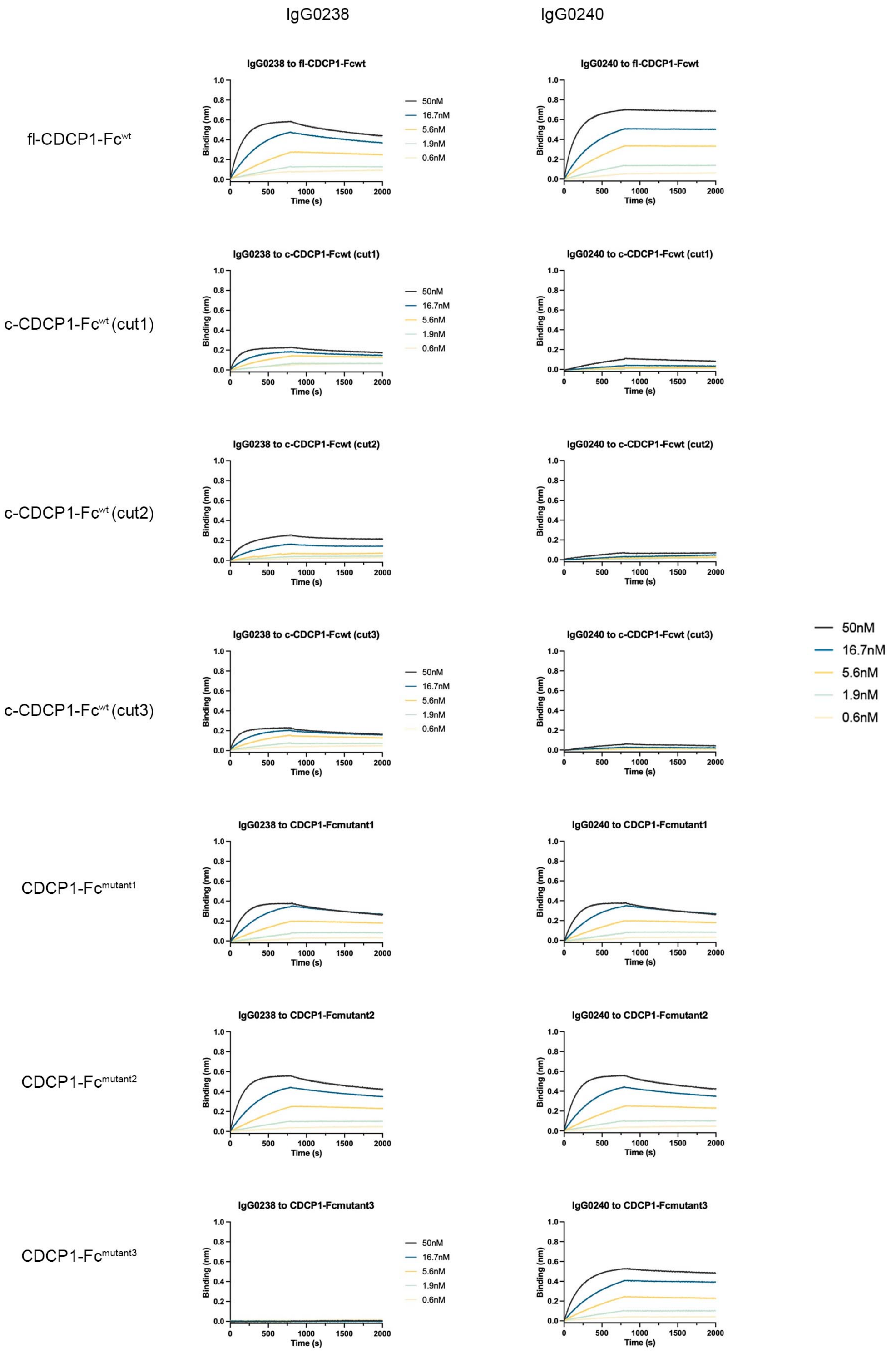

SI

Appendix, Figure 2. BLI characterization of IgG0151, 0152, 0153, 0238, and 0240 binding to uncleaved CDCP1, cleaved CDCP1 with different cut sites and three CDCP1 mutants used for negative selection.

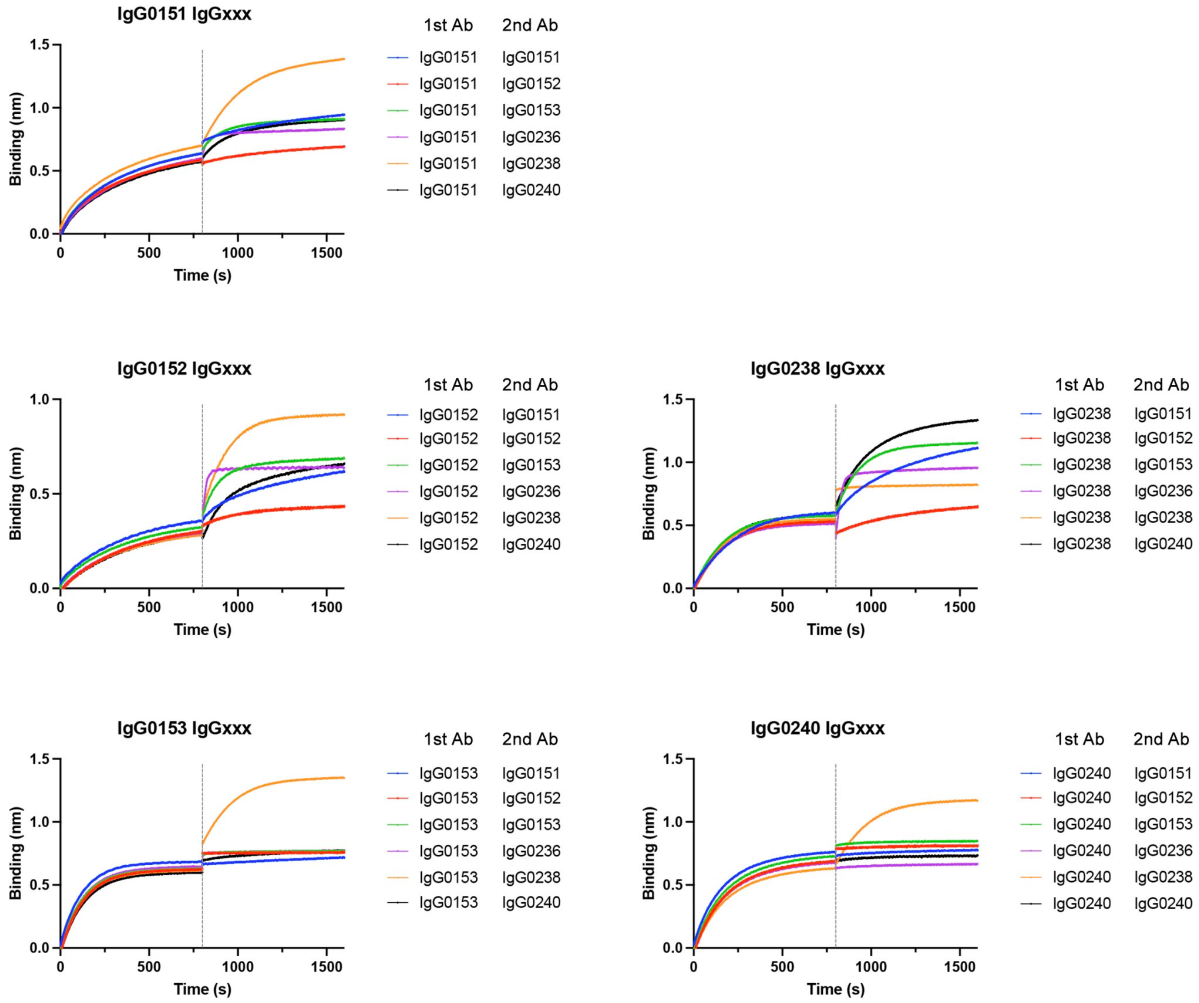

SI Appendix, Figure 3. Epitope binning competition experiments by a sequential binding using BLI. The IgG concentration used for assay is 100 nM.

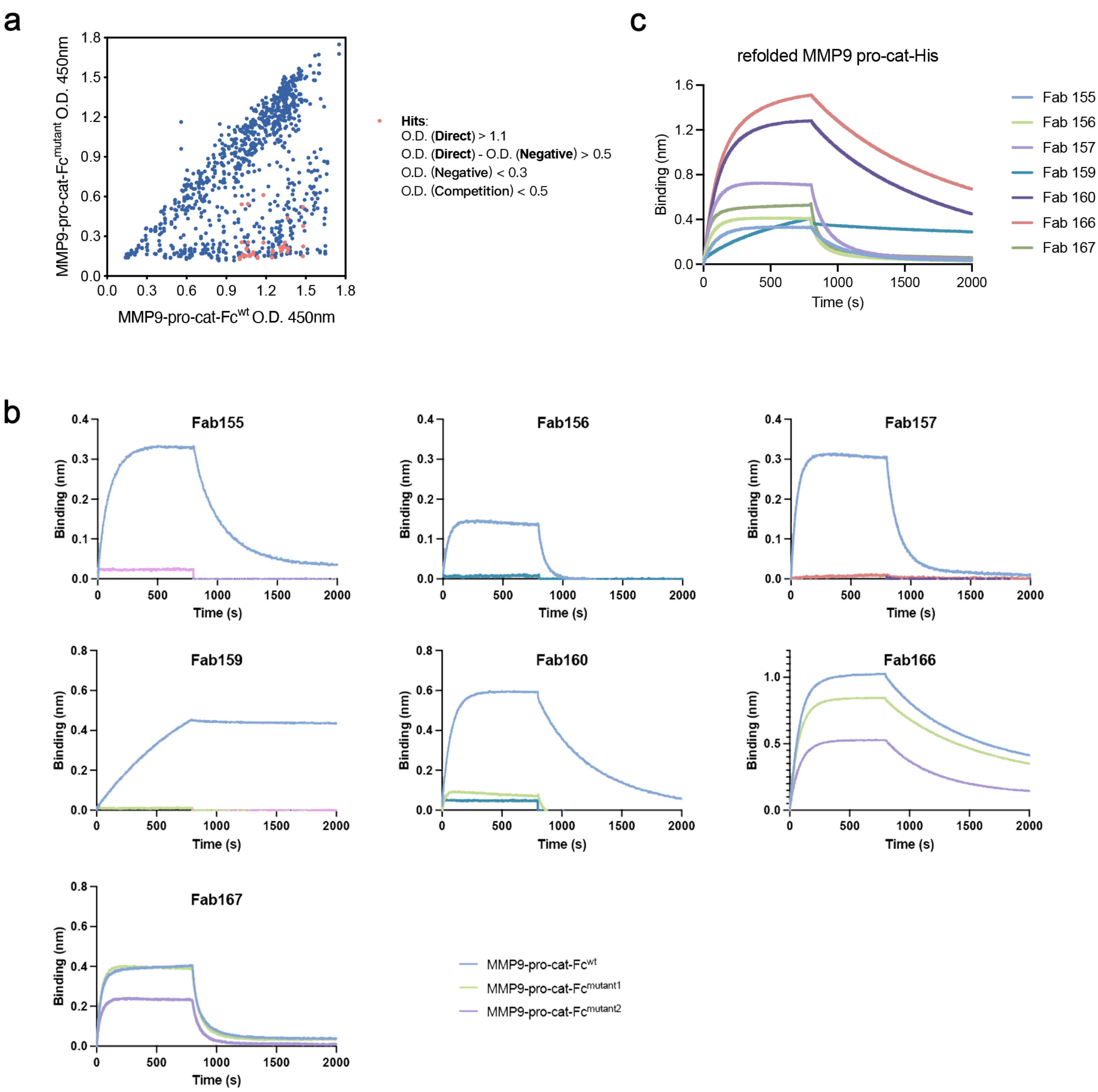

SI Appendix, Figure 4. (a) Screening of binding of Fab phage clones to MMP9-pro-cat-Fc^{wt} and MMP9-pro-cat-Fc^{mutant1or2} by single point phage ELISA. Hits (red dots) are defined as those that preferentially bind to WT over decoys, are easily competed off with soluble MMP9-pro-cat-Fc^{wt}, and exhibit minimum nonspecific binding. O.D. values for Direct, Negative, Competition, and BSA binding are defined in **SI Appendix, Fig. 1c**. (b) BLI characterization of different Fabs. (c) BLI characterization confirmed the binding of these seven Fabs to truncated WT MMP9 with His-tag (MMP9-pro-cat-His^{WT}) expressed and purified from E coli inclusion bodies.

SI Appendix, Table 1.

Target	Positive selection antigen	Negative selection antigen	Mutation made	Fold of enrichment
**MMP1**	MMP1-pro-cat-Fc^{wt} (aa20-259)	MMP1-pro-cat-$Fc^{mutant1}$	QF(99-100) -> AA	~10
		MMP1-pro-cat-$Fc^{mutant2}$	VAQFVL (97-102) -> GGSGGS	~100
**MMP3**	MMP3-pro-cat-Fc^{wt} (aa39-215_aa391-444)	MMP3-pro-cat-$Fc^{mutant1}$	HF (99-100) -> AA	~10
		MMP3-pro-cat-$Fc^{mutant2}$	VGHFRT (97-102) -> GGSGGS	~100
**MMP9**	MMP9-pro-cat-Fc^{wt} (aa39-215_aa391-444)	MMP9-pro-cat-$Fc^{mutant1}$	RF(106-107) -> AA	~10
		MMP9-pro-cat-$Fc^{mutant2}$	LGRFQT(104-109) -> AGAAAA	~100

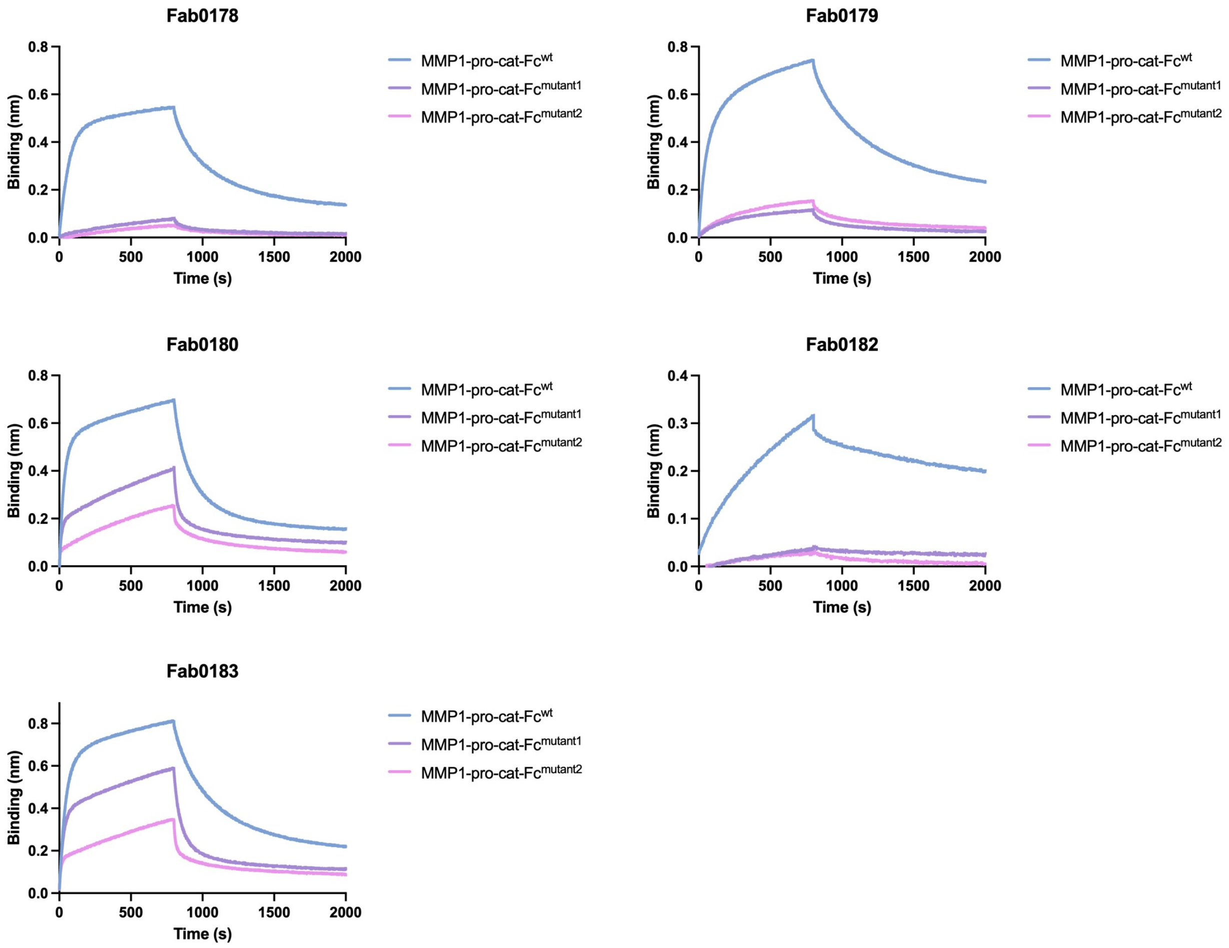

SI Appendix, Figure 5. BLI characterization of Fabs from selection campaign for MMP1. These Fabs selectively or preferentially bind to WT antigens over at least one of the two EOI decoys.

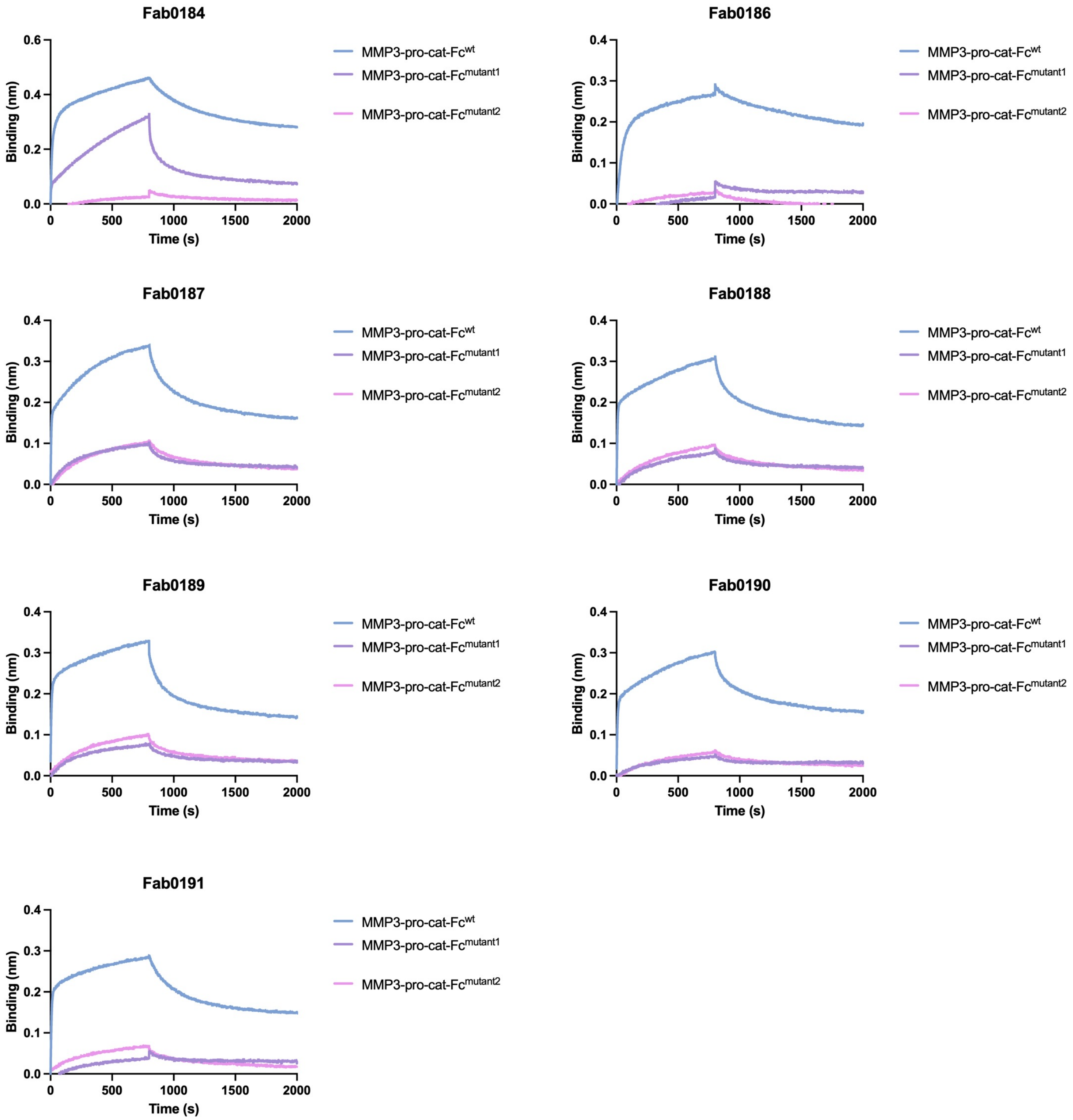

SI Appendix, Figure 6. BLI characterization of Fabs from selection campaign for MMP3. These Fabs selectively or preferentially bind to WT antigens over at least one of the two EOI decoys.

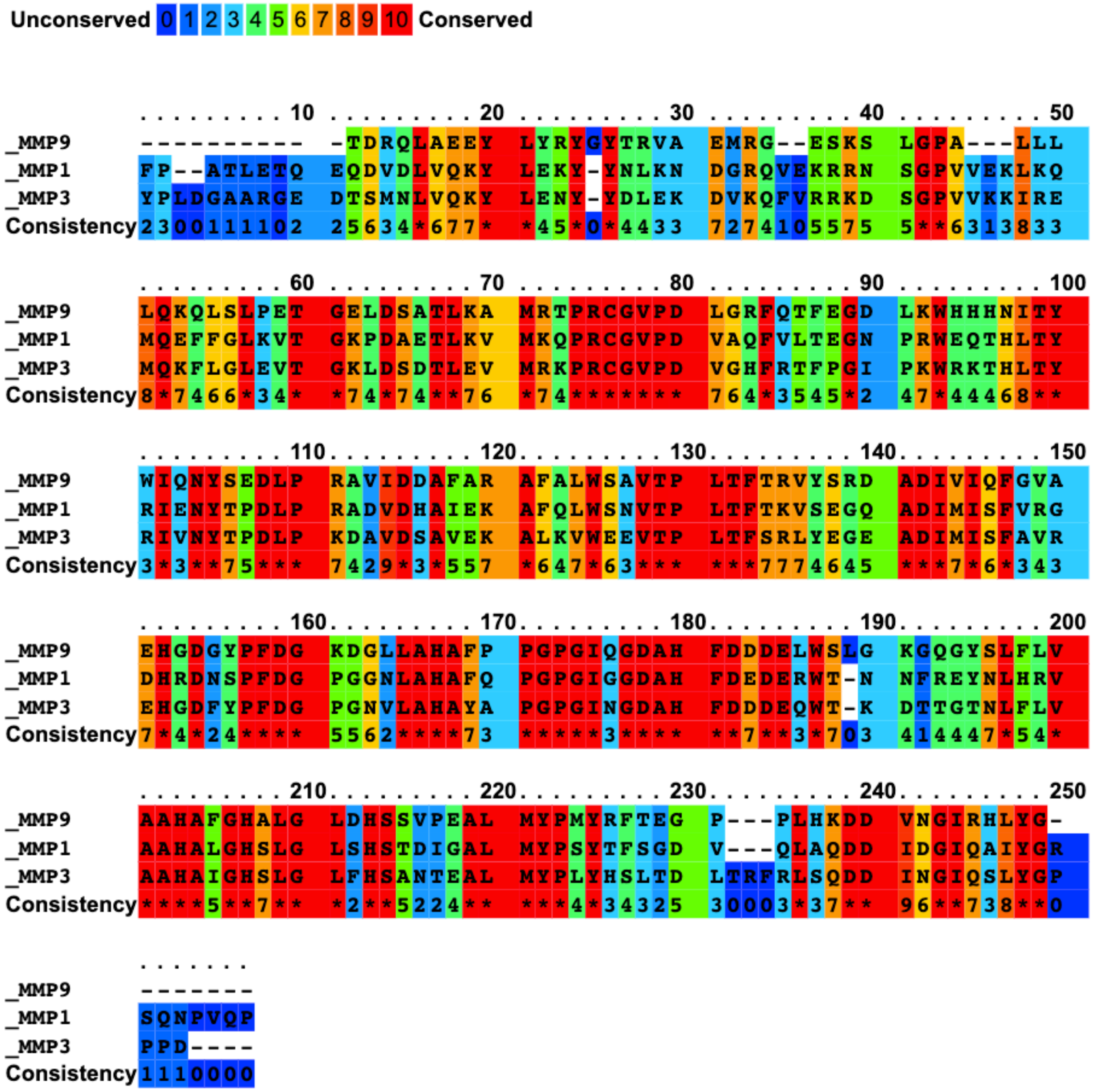

SI Appendix, Figure 7. Sequence alignments of pro or active MMP1, 3, and 9.

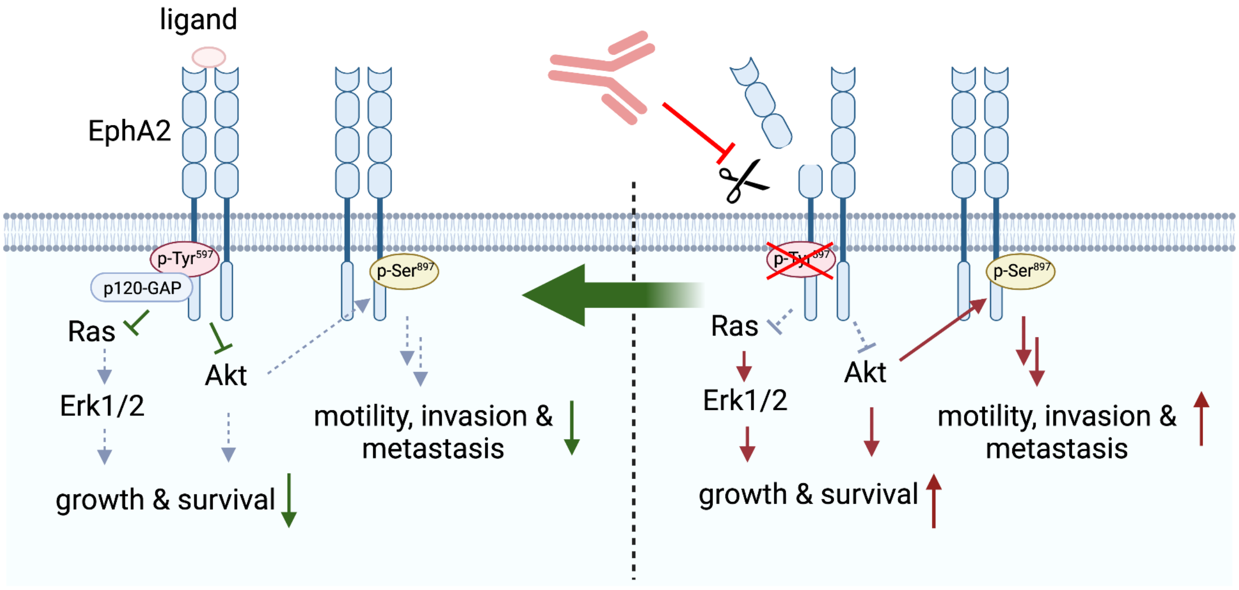

SI Appendix, Figure 8. Therapeutic potential of EphA2 proteolysis blocking antibody.

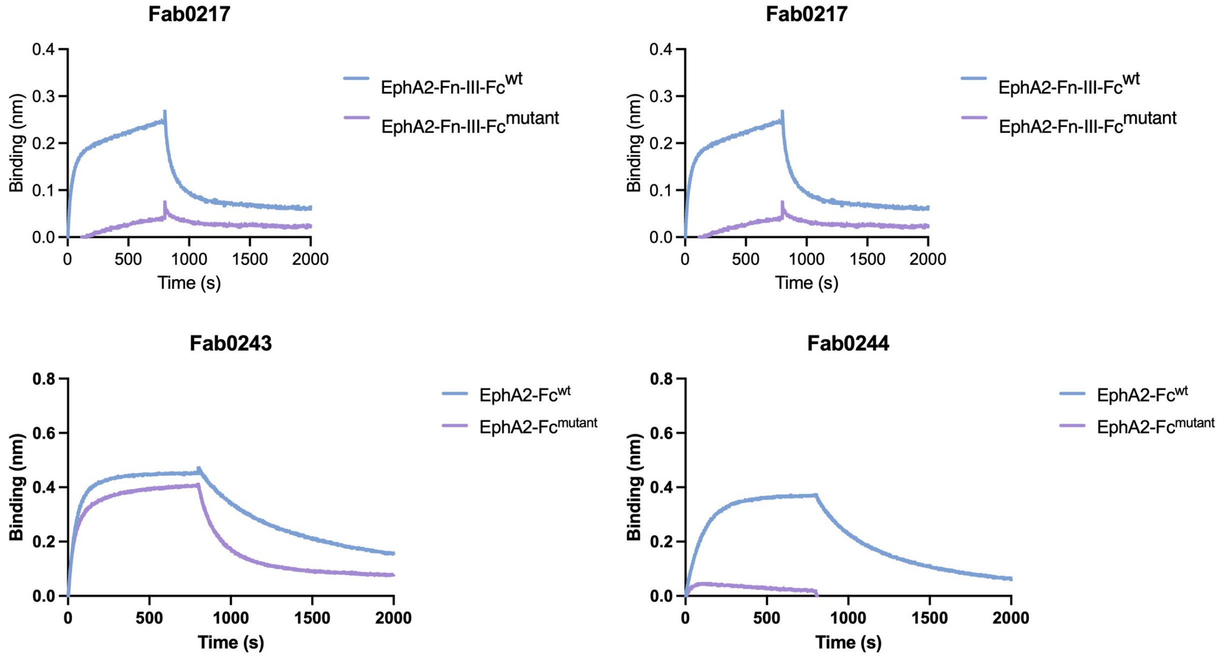

SI Appendix, Figure 9. BLI characterization of Fabs from selection campaign for EphA2. These Fabs selectively or preferentially bind to WT antigens over the EOI decoys.

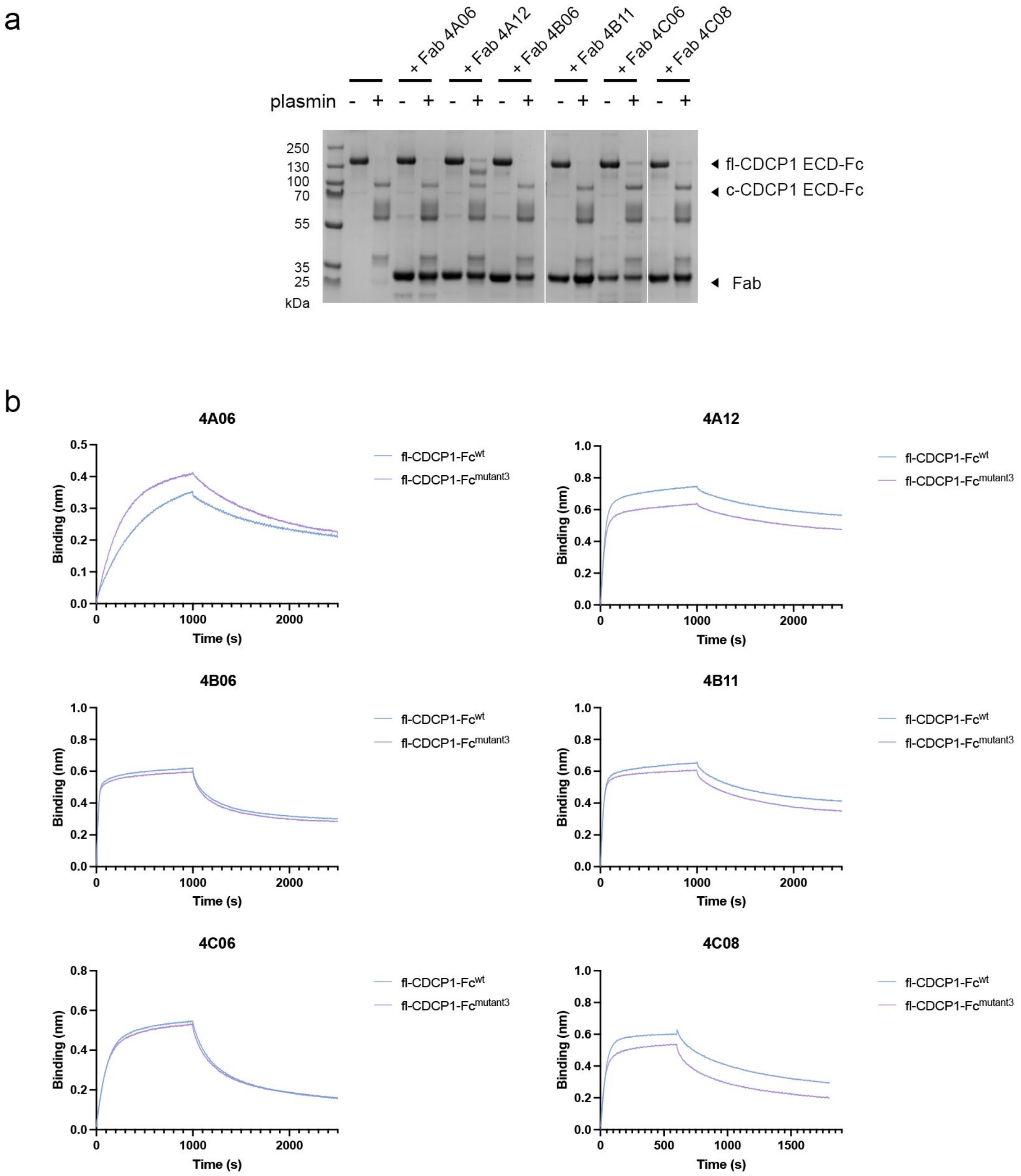

SI Appendix, Figure 10. (a) The anti-CDCP1 Fabs, derived from a phage selection campaign employing the Fc domain for negative selection and full-length CDCP1 ECD-Fc for positive selection, do not target the proteolytic sites of CDCP1. As a result, these Fabs are unable to inhibit the proteolysis of CDCP1 by plasmin. (b) These anti-CDCP1 Fabs bind to both CDCP1 WT and mutant3.
